# Peri-arrest ventilation with positive end-expiratory-pressure vs. zero end-expiratory-pressure in out-of-hospital-cardiac-arrest (PerAVent)—a prospective, cluster-randomized multicenter trial

**DOI:** 10.1186/s13063-025-09292-w

**Published:** 2026-01-21

**Authors:** Mathini Vaseekaran, Tobias Vollmer, Lydia Johnson Kolaparambil Varghese, Christina Zeichen, Jochen Hinkelbein, Raphael Abels, Bernd Strickmann, Martin Deicke, Julia Johanna Grannemann, Rainer Grünzig, Annika Hoyer, Jan Persson, Jens Tiesmeier, Gunter Veit, Gerrit Jansen

**Affiliations:** 1 Johannes Wesling Hospital Minden, University Clinic for Anaesthesiology, Intensive Care Medicine, Emergency Medicine and Pain Medicine, Ruhr-University Bochum, Hans-Nolte-Straße 1, Minden, 32429 Germany; 2 Emergency Medical Service District of Guetersloh, District of Gütersloh, Gütersloh, 33324 Germany; 3 Emergency Medical Service District of Osnabrueck, Am Schoelerberg 1, Osnabrueck, 49082 Germany; 4 Biostatistics and Medical Biometry, Medical School OWL, Bielefeld University, Universitätsstraße 25, Bielefeld, 33605 Germany; 5 Krankenhaus Luebbecke, Clinic for Anaesthesiology, Intensive Care Medicine and Emergency Medicine, Virchowstr. 65, Lübbecke, 32312 Germany; 6 Emergency Medical Service District of Minden-Luebbecke, District of Minden-Luebbecke, Rechts- und Ordnungsamt, Feuerwehrtechnisches Zentrum (FTZ), Eickhorster Str. 33, Hille, 32479 Germany

**Keywords:** Cardiac arrest, Positive end-expiratory pressure, Ventilation, Randomized controlled trial, Emergency medicine, Resuscitation, CPR

## Abstract

**Background:**

Out-of-hospital cardiac arrest (OHCA) remains a major cause of mortality, with low survival probabilities to hospital discharge. Despite the frequent use of airway management and mechanical ventilation during resuscitation, there is limited evidence regarding the optimal ventilation strategy to improve oxygen delivery and patient outcomes. The present study aims to investigate the effects of positive-end-expiratory-pressure (PEEP) set at 5 mbar compared to zero-end-expiratory-pressure (ZEEP) on the return of spontaneous-circulation (ROSC) in adult patients with OHCA.

**Methods:**

This is a prospective, multicenter, cluster-randomized controlled trial conducted across emergency medical services (EMS) in the regions of Gütersloh, Minden-Lübbecke, and Osnabrück. Adult patients (≥ 18 years) with OHCA who are undergoing mechanical ventilation through an airway device will be enrolled. The clusters (regional districts) will be randomized into two groups: one group will receive ventilation with PEEP set at 5 mbar (intervention group), while the other group will receive ventilation with ZEEP (control group). The study’s primary endpoint is the occurence of ROSC. Secondary endpoints include occurence of re-arrest, death during pre-hospital care phase, hospital admission during ongoing resuscitation, hospital admission with spontaneous circulation, peripheral oxygen saturation, and endtidal CO_2_ at hospital admission.

**Discussion:**

Optimal ventilation strategies during OHCA have not been well established. The use of PEEP may improve oxygenation and oxygen delivery. This study aims to provide crucial data on whether the application of PEEP at 5 mbar vs. 0 mbar can improve the probability of ROSC without adversely affecting hemodynamics. The findings could inform future guidelines on ventilation strategies in resuscitation.

**Trial registration:**

The trial is registered on ClinicalTrials.gov under the registration number NCT06836830. 24.02.2025 https://clinicaltrials.gov/study/NCT06836830?term=gerrit%20jansen&rank=2.

## Administrative information

**Table:** 

Title {1}	Peri-Arrest Ventilation with Positive End-Expiratory-Pressure vs. Zero End-Expiratory-Pressure in Out-of-Hospital-Cardiac-Arrest (PerAVent) – a prospective, cluster-randomized multicenter trial
Trial registration {2a and 2b}	The trial is registered on ClinicalTrials.gov under the registration number NCT06836830. 24.02.2025 https://clinicaltrials.gov/study/NCT06836830?term=gerrit%20jansen&rank=2
Protocol version {3}	Date: 21.10.2024 Version 1.0
Funding {4}	To conduct study meetings, create a study website, develop a study logo, provide the existing network for potential further recruitment of study centers, and promote the study within the German Society for Anaesthesiology, Intensive Care Medicine, Emergency Medicine, and Pain Therapy (DGAI) at conferences, a funding application was submitted to the DGAI Study Center. The DGAI Study Center provides a one-time grant of € 15,000. In addition, the study is supported by the hospital's own resources and the resources of the participating rescue service areas
Author details {5a}	Priv.-Doz. Dr. med. Gerrit Jansen, MHBA Johannes Wesling Hospital Minden University hospital for anaesthesiology, intensive care medicine, emergency medicine and pain medicine Ruhr-University of Bochum Hans-Nolte-Straße 1 32,429 Minden Germany Tel. 0571/7 90–5 4404 gerrit.jansen@muehlenkreiskliniken.de
Name and contact information for the trial sponsor {5b}	Johannes Wesling Hospital Minden University hospital for anaesthesiology, intensive care medicine, emergency medicine and pain medicine Ruhr-University of Bochum Hans-Nolte-Straße 1 32,429 Minden Germany

## Introduction

### Background and rationale {6a}

Out-of-hospital cardiac arrest (OHCA) occurs in approximately 67 to 170 individuals per 100,000 inhabitants annually across Europe, with survival to hospital discharge ranging from 8 to 18% [1]. Despite advances in resuscitation science, overall outcomes remain poor. Early, high-quality chest compressions and timely defibrillation remain key determinants of prognosis.

While the importance of optimal airway management during cardiopulmonary resuscitation (CPR) has been extensively studied and remains under debate, the evidence base for optimal ventilation strategies—particularly the application of positive end-expiratory pressure (PEEP) during CPR—is sparse.

In contrast, numerous studies in intensive care medicine (ICU) [2–4] have demonstrated that PEEP improves oxygenation, alveolar recruitment, and overall pulmonary mechanics [5–7]. However, the physiological conditions during cardiac arrest differ markedly from those in the ICU, especially with respect to circulation, intrathoracic pressures, and venous return.

Therefore, the transferability of established ICU findings to the resuscitation setting remains uncertain, and direct clinical evidence is limited [3, 4, 6, 8]. Investigating whether modest levels of PEEP during OHCA can improve oxygen delivery and alveolar recruitment without compromising hemodynamics is thus of high clinical relevance and may help bridge the gap between intensive care ventilation strategies and resuscitation practice.

Guidelines from the European Resuscitation Council provide general recommendations for ventilation during cardiac arrest but do not address the specific application of PEEP in this context [8]. Although every secured airway inevitably requires ventilation afterwards, there is little evidence supporting the best approach to ventilation after airway management.

Regarding whether manual ventilation using a bag-valve device or mechanical ventilation is more beneficial, there is currently limited evidence but many subjective opinions. Some individuals argue that having a tactile sense of lung compliance during manual ventilation can be advantageous, while other studies (e.g., from the Belgian Registry) suggest that mechanical ventilation after the insertion of an airway device might be beneficial. Despite these differing perspectives, there is still limited data on optimal ventilation settings.

For instance, one study demonstrated that a ventilation rate of 10 to 20 breaths per minute with controlled tidal volumes of 6 ml/kg and 100% oxygen during CPR resulted in ROSC in approximately 50% of cases, significantly higher than the usual ROSC probabilities of ≤ 30% [6].

As known from intensive care medicine PEEP can improve oxygenation by increasing functional residual capacity and recruiting collapsed alveoli, potentially benefiting patients undergoing CPR. However, PEEP may also reduce venous return and cardiac output, which could adversely affect the outcome. Therefore, determining the optimal PEEP level is critical in balancing the benefits of improved oxygenation against the risks of decreased cardiac output [8].

Existing studies have shown mixed results regarding the impact of PEEP during CPR. Animal studies have suggested that PEEP levels between 0 and 5 mbar may optimize the balance between oxygen delivery and hemodynamic stability. Specifically, a PEEP of 5 mbar was associated with the highest oxygen delivery and minimal adverse effects on cardiac output. However, PEEP levels above 10 mbar were shown to significantly reduce cardiac output [7].

The aim of the present study is to determine if a PEEP of 5 mbar during OHCA improves the likelihood of ROSC compared to ZEEP.

### Objectives {7}

The objective of the present study is to evaluate the effect of PEEP of 5 mbar vs. 0 mbar on the occurence of ROSC in OHCA.

### Trial design {8}

The study is designed as a prospective, cluster-randomized, multicenter interventional pilot trial. It employs a cluster-randomized design, where each participating EMS region in pre-defined time frames represents a cluster. The clusters will be randomly allocated to either the intervention group (receiving a PEEP of 5 mbar) or the control group (receiving ZEEP or 0 mbar) for a defined period of 6 months, followed by a crossover, where the same regions will switch to the opposite treatment arm for another 6 months.

The allocation ratio is 1:1 within each cluster, meaning that each group (intervention and control) will receive an equal number of patients. Since this is a pilot study, it is designed primarily to assess the feasibility and preliminary effects of the intervention rather than to definitively establish superiority. The results will provide the groundwork for future, more extensive studies.

## Methods: participants, interventions and outcomes

### Study setting {9}

The study will be conducted in pre-hospital EMS settings across multiple regions in Germany. Specifically, the study will involve EMS units from the following districts:

- Guetersloh
- Minden-Luebbecke
- Osnabrueck

With approximately 650,000 inhabitants across all regions combined. These EMS regions serve as the primary sites for data collection, and all resuscitation efforts in OHCA cases in these areas will be included. The study focuses on real-world pre-hospital settings where EMS teams provide critical care during cardiac arrest events before hospital admission.

### Eligibility criteria {10}

#### Inclusion

- Adults ≥ 18 years
- Non-traumatic OHCA
- Mechanical ventilation via airway device

#### Exclusion

- Patients < 18 years
- Traumatic cause of OHCA
- No cardiac arrest
- Withholding of resuscitation (e.g., Do-not-Resuscitate orders).

### Additional considerations

Variables such as difficulty in airway management, timing of OHCA in relation to EMS arrival, and pregnancy will not serve as exclusion criteria.

These parameters are systematically documented in the standardized electronic case report form (eCRF) for every resuscitation case, allowing exploratory subgroup analyses to assess their potential influence on the primary outcome (ROSC).

Cases of clearly irreversible death (e.g., rigor mortis, decapitation, or other obvious signs of death) are not eligible for resuscitation and therefore not included in the study, in line with current resuscitation guidelines.

### Who will take informed consent? {26a}

Due to the nature of this study’s setting in a time-sensitive emergency situation and the current valuation of both treatment options, no consent is necessary. As every patient receives therapy in line with the guidelines, this was confirmed and approved by the ethics committee of the Ruhr-University Bochum (file reference 2024–1148) on 09.01.2024.

### Additional consent provisions for collection and use of participant data and biological specimens {26b}

Due to the nature of this study’s setting in a time-sensitive emergency situation and the current valuation of both treatment options, no consent is necessary. This was confirmed and approved by the ethics committee of the Ruhr-University Bochum (file reference 2024–1148) on 09.01.2024.

## Interventions

### Explanation for the choice of comparators {6b}

The choice of comparators in this study—namely PEEP at 5 mbar versus ZEEP at 0 mbar—is based on current gaps in clinical evidence regarding optimal ventilation strategies during OHCA. While mechanical ventilation during CPR is a critical component of patient care, there is limited data on the impact of varying levels of PEEP on outcomes such as ROSC.

PEEP has the potential to improve oxygenation by increasing functional residual capacity and recruiting collapsed alveoli, improving oxygen delivery to the tissues which could enhance the chances of ROSC [5]. However, higher levels of PEEP may also reduce venous return and subsequently cardiac output, which could be detrimental during resuscitation [7].

By comparing PEEP at 5 mbar (which animal studies suggest optimizes oxygen delivery without compromising hemodynamics) with ZEEP (the standard of care in many EMS settings), this study aims to determine whether the use of a modest level of PEEP can enhance resuscitation outcomes. Studies have indicated that a PEEP of 5 mbar may offer the best balance between oxygenation and maintaining cardiac output, which is crucial during resuscitation [7].

ZEEP was chosen as the comparator because it represents the baseline condition where no additional pressure is applied during the expiratory phase. This is a common practice in pre-hospital settings, where the main goal is to avoid excessive pressures that might negatively impact circulation [8]. Will the use of a moderate level of PEEP during resuscitation ultimately prove more effective compared to using ZEEP in improving patient outcomes during OHCA?

### Intervention description {11a}

The study will involve two groups of patients experiencing OHCA:

- PEEP Group (Intervention Group): Patients will receive mechanical ventilation with PEEP set at 5 mbar using the Medumat Transport ventilator, which includes an integrated PEEP valve allowing standardized settings. This aims to improve oxygenation during cardiopulmonary resuscitation (CPR).
- ZEEP Group (Control Group): Patients will receive mechanical ventilation with ZEEP (0 mbar) using the same ventilator model, representing the current standard practice in many EMS systems.

In the participating emergency medical services, PEEP application via manual bag-valve-mask ventilation is not intended and is not routinely performed in adult patients. Therefore, all PEEP interventions within this study are delivered exclusively via mechanical ventilation using the Medumat Transport device.

The specific ventilation settings (PEEP or ZEEP) are pre-assigned according to the cluster randomization and will be maintained throughout the resuscitation phase unless the treating physician deems a change clinically necessary.

### Criteria for discontinuing or modifying allocated interventions {11b}

The ventilation settings (PEEP or ZEEP) may be modified if the emergency team, based on their clinical judgment during the intervention, deems it necessary to make changes.

### Strategies to improve adherence to interventions {11c}

To ensure adherence to the intervention protocols, the following strategies will be implemented:

- Training and regular updates for all emergency medical personnel involved in the study.
- Standardized protocols for the use of ventilators and documentation of ventilation settings.
- Continuous monitoring and recording of ventilation parameters by the ventilator devices to ensure compliance.
- Periodic reviews of recorded data to identify and correct any deviations from the protocol.

These measures aim to maintain consistency across all trial sites and ensure adherence to the assigned interventions.

### Relevant concomitant care permitted or prohibited during the trial {11d}

All standard resuscitation measures according to current European Resuscitation Council (ERC) guidelines are permitted, including chest compressions, defibrillation, and administration of emergency medications (e.g., epinephrine, amiodarone, lidocaine).

After ROSC, ventilation management will follow standardized targets across both study groups to ensure consistency during the post-resuscitation phase:

- FiO₂ titration to maintain peripheral oxygen saturation (SpO₂) above 94%
- End-tidal CO₂ (ETCO₂) between 35 and 45 mmHg
- Tidal volume of 6–8 mL/kg predicted body weight

The PEEP level will remain consistent with the group allocation throughout the post-ROSC phase—5 mbar in the intervention group and 0 mbar (ZEEP) in the control group—as documented in the eCRF.

This approach ensures uniform post-ROSC ventilation management while maintaining the integrity of the randomized intervention. All parameters are automatically recorded and subsequently extracted from the ventilator and eCRF for analysis.

### Provisions for post-trial care {30}

The study concludes upon the patient’s admission to the hospital.

### Outcomes {12}

- Primary outcome:

The primary outcome is return of spontaneous circulation (ROSC), defined according to the Utstein template as the presence of a palpable carotid or femoral pulse during or after resuscitation, optionally confirmed by Doppler or ultrasound when available [9].

ROSC will be measured as a binary variable (yes/no), and the analysis will compare the proportion of patients achieving ROSC between the two study groups (PEEP vs. ZEEP).

This definition ensures consistency with international reporting standards and enhances the reliability of outcome assessment.

- Secondary outcomes:
  o Re-Arrest-Rate
  o Death during pre-hospital care phase
  o Hospital admission during ongoing resuscitation
  o Hospital admission with spontaneous circulation
  o SpO_2_ at hospital admission
  o FiO_2_ at hospital admission
  o etCO_2_ at hospital admission
- Other outcomes:
  o Date of death (if applicable), analyzed as a time-to-event outcome, focusing on survival duration post-resuscitation.
  o Date of hospital discharge for survivors, measured as time to discharge.
  o Neurological status, measured as the Cerebral Performance Category at hospital discharge.

These outcomes are relevant as they provide a comprehensive assessment of both immediate and longer-term clinical effects of the interventions. ROSC is vital for short-term survival, while neurological status and vital signs reflect the quality of resuscitation and longer-term outcomes. Time to death and discharge further provide insight into survival and recovery rates.

### Participant timeline {13}

#### Enrolment

**Fig. 1:**
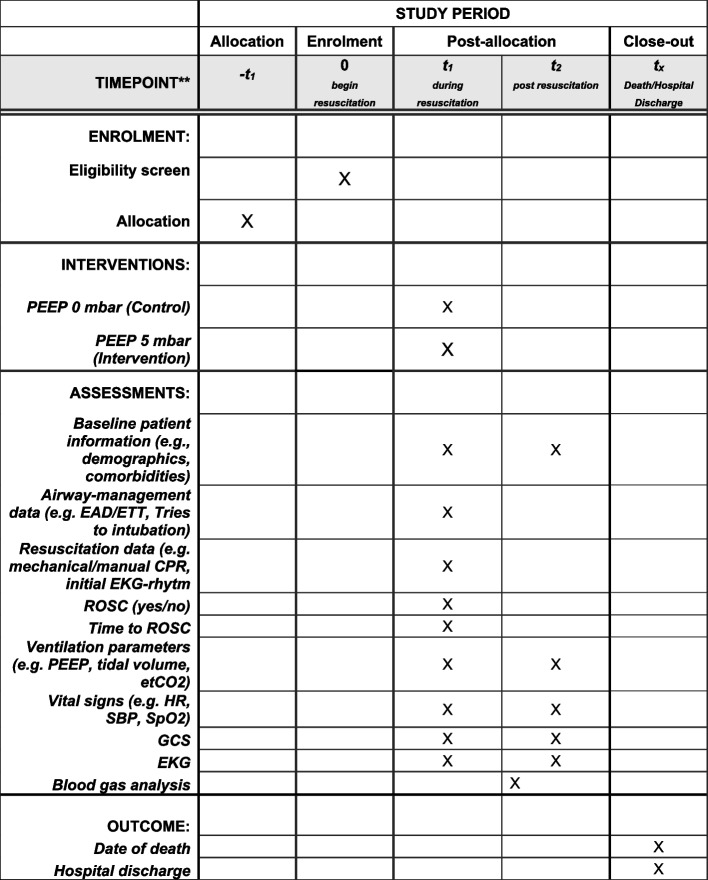
Example template of recommended content for the schedule of enrolment, interventions, and assessments. Recommended content can be displayed using various schematic formats. See SPIRIT 2013 Explanation and Elaboration for examples from protocols. **List specific timepoints in this row

- *Time Point*: Patients will be enrolled *at the time of OHCA *(Fig. 1).
- *Inclusion Process*: Once a patient meets the inclusion criteria (OHCA, age ≥ 18 years, and undergoing mechanical ventilation), they will be immediately enrolled by the EMS team. The need for informed consent was waived by the responsible ethical committee.

#### Interventions

- PEEP or ZEEP Intervention:
  o *Time Point*: Immediately after advanced airway management is secured (using either an extraglottic airway device [EAD] or an endotracheal tube [ETT]).
  o The assignment to PEEP or ZEEP is predetermined, as the defined time period dictates whether the patient will receive PEEP (5 mbar) or ZEEP (0 mbar). The sequence of these time periods has been randomized via cluster randomization.
  o The intervention will continue throughout the resuscitation efforts until ROSC or cessation of resuscitation efforts.

### Assessments

- During resuscitation:
  o Continuous monitoring of vital signs (e.g., HR, SpO_2_) and ventilation parameters (e.g., PEEP, tidal volume, etCO_2_).
- Post-resuscitation:
  o *Time Point*: Immediately upon achieving ROSC until handover at the hospital.
  o *Measurements*: GCS, vital signs (HR, SpO_2_), and optional initial blood gas analysis will be assessed.
- Follow-up assessments:
  o *Time Point*: During hospital stay, including assessments of neurological outcomes and cardiac function (e.g., EKG, vital signs) if applicable.
  o *Final Assessment*: Date of death (if applicable) or hospital discharge.

### Visits for participants

- No formal follow-up visits are required post-resuscitation as all data will be collected during the pre-hospital and hospital phases. The study focuses on immediate outcomes following OHCA and does not include long-term follow-up.

### Sample size {14}

The planned sample size is approximately 600 out-of-hospital cardiac arrest (OHCA) cases, with about 300 patients per group (PEEP vs. ZEEP).

The PerAVent trial is designed as a pilot and feasibility study, primarily aimed at evaluating the practical implementation of PEEP versus ZEEP ventilation in the pre-hospital setting and generating preliminary data for future confirmatory research.

The study hypothesis is that ventilation with PEEP (5 mbar) increases the probability of return of spontaneous circulation (ROSC) compared with ZEEP (0 mbar).

As this is a pilot study, no formal sample size calculation or power analysis has been performed, since no reliable prior data exist on which to base such assumptions. The chosen sample size is therefore based on feasibility considerations and expected case numbers within the participating emergency medical service regions over the planned study period.

The collected data will serve to estimate key parameters—such as recruitment rates, adherence, and effect size estimates—that may inform the design of a future confirmatory trial.

### Recruitment {15}

Recruitment will occur across multiple EMS regions, including Gütersloh, Minden-Lübbecke, and Osnabrück. All eligible OHCA patients treated by EMS teams in these regions during the 12-month data collection period will be enrolled. The large geographic area and population served by these EMS units are expected to provide sufficient participant numbers.

## Assignment of interventions: allocation

### Sequence generation {16a}

In this study, the allocation sequence will be generated using *cluster randomization*. Each participating EMS region (Gütersloh, Minden-Luebbecke, and Osnabrueck) will represent a cluster. The clusters will be randomized to either the intervention group (PEEP) or the control group (ZEEP) using *predefined randomization blocks*, which are based on 6-month intervals. After 6 months, the clusters will switch to the opposite group, ensuring that each region experiences both interventions.

To reduce predictability and maintain the integrity of the randomization, the allocation sequence and any randomization restrictions, such as block sizes, will be managed centrally and will not be accessible to the EMS personnel enrolling participants or assigning interventions. This ensures that the allocation remains concealed until implementation.

### Concealment mechanism {16b}

The allocation sequence will be implemented through a centralized randomization process. The sequence will be concealed from the EMS personnel by utilizing pre-determined randomization blocks that are centrally managed. EMS personnel will not have access to the allocation until the intervention is assigned ensuring allocation concealment throughout the enrolment process. Through regular training sessions, participants will be regularly informed about the assignment within the corresponding time period.

### Implementation {16c}

The allocation sequence will be generated by the study’s central coordinating team. EMS personnel in the participating regions (Gütersloh, Minden-Luebbecke, and Osnabrueck) will be responsible for enrolling participants based on eligibility criteria. However, the actual assignment of participants to the PEEP or ZEEP group will occur automatically according to the pre-determined allocation sequence managed by the central team.

## Assignment of interventions: blinding

### Who will be blinded {17a}

In this study, the EMS personnel delivering the interventions (PEEP or ZEEP) will not be blinded, as they need to know which ventilation strategy to apply during resuscitation. Since the patients enrolled in the study are unaware of which group they are assigned to during the resuscitation, they are considered blinded.

However, the outcome assessors and data analysts who evaluate the results, including ROSC probabilities and other clinical outcomes, will be blinded to the group assignments to minimize bias in data interpretation and analysis.

This blinding ensures that the analysis is conducted without any knowledge of which intervention was applied, maintaining objectivity in assessing the study’s outcomes.

### Procedure for unblinding if needed {17b}

Not applicable.

## Data collection and management

### Plans for assessment and collection of outcomes {18a}

Data acquisition will be carried out using an eCRF, which is to be completed by the emergency care team following the resuscitation. The data collected will be supplemented with the ventilation parameters recorded by the emergency ventilators. The variables collected in the eCRF are shown in Table 1.

**Table 1:** Variables collected in the eCRF

Category	Collected variables
Patient factors	Age (years), Sex, Comorbidities
Airway factors	Bag-mask ventilation before airway device placement (yes/no), Intubation (extraglottic airway device vs. endotracheal tube), Type of extraglottic airway device, Number of insertion attempts, Complications during insertion, Number of intubation attempts, Complications during intubation, Abandonment of intubation attempts, Re-intubation by emergency physician
Resuscitation data	Observed cardiac arrest (yes/no), Cardiac arrest observed by (layperson/emergency services), Layperson CPR performed (yes/no), Time to initiation of chest compressions, Resuscitation duration (minutes), Initial ECG findings, Defibrillation (yes/no), Number of defibrillations, Type of chest compressions (manual vs. mechanical), Type of mechanical compression device, Administration and cumulative dose of epinephrine and amiodarone
Intra-arrest ventilation data	Ventilation parameters set by emergency services (Pmax, PEEP, respiratory rate, tidal volume, inspiration-expiration ratio, FiO_2_, Ventilation mode: IPPV, BILevel, CCSV, Other), Ventilation parameters measured by Medumat Transport, Changes in ventilation parameters during resuscitation
Post-arrest treatment	Re-intubation from extraglottic airway to endotracheal tube (yes/no), Number of intubation attempts, Type and dose of analgesic, hypnotic, muscle relaxant, Complications in pre-hospital post-arrest phase (aspiration, ventilation issues, hypotension, re-arrest), Administration of catecholamines, Post-arrest ventilation settings
Additional outcome data	Consciousness at handover, ECG findings at handover, Glasgow Coma Scale, Vital parameters at handover (systolic blood pressure, heart rate, oxygen saturation, respiratory rate, etCO_2_), Initial blood gas analysis, Date of death, Hospital discharge date

### Plans to promote participant retention and complete follow-up {18b}

Not applicable.

### Data management {19}

Data from the study will be entered and managed using a centralized eCRF provided by the study center of the University of Bonn, which allows access to a for all study participants. All patient data will be recorded both in the eCRF and as part of the standard EMS documentation process.

To ensure data quality, several measures will be implemented. Data will be systematically coded to ensure consistency across all entries, with patient identifiers anonymized to protect confidentiality. All data entered into the app will be encrypted and securely stored in a centralized database, accessible only to authorized study personnel.

### Confidentiality {27}

Personal information about potential and enrolled participants will be handled with strict confidentiality at all stages of the trial. The following measures will be taken to ensure data protection. Participant data, including personal identifiers, will be collected via secure electronic systems, such as the eCRF provided by the University of Bonn. During data entry, personal identifiers will be anonymized, and participants will be assigned unique study IDs to ensure confidentiality. Access to identifiable information will be limited to authorized personnel only. Study data shared with external parties or for analysis purposes will be fully de-identified, ensuring no participant can be traced back using the shared information. All data will be stored on secure, encrypted servers, both during the trial and after its completion. Physical copies of documents, if needed, will be kept in locked, access-controlled storage. Data will be retained according to regulatory requirements and then properly disposed of to ensure no breach of confidentiality. Additionally, participants are subject to the legal data protection regulations.

### Plans for collection, laboratory evaluation and storage of biological specimens for genetic or molecular analysis in this trial/future use {33}

Not applicable.

## Statistical methods

### Statistical methods for primary and secondary outcomes {20a}

For the primary outcome (ROSC), a mixed logistic regression model will be used to analyze the difference in the probability of achieving ROSC between the two groups (PEEP vs. ZEEP). This model will account for the cluster-randomized design, adjusting for potential correlations within clusters (EMS regions).

For secondary outcomes, such as re-arrest rate, death during pre-hospital care, hospital admission during ongoing resuscitation, hospital admission with spontaneous circulation, SpO₂, FiO₂, and etCO₂ at hospital admission, linear regression models will be used for continuous variables, while logistic regression will be applied for binary outcomes (e.g., shockable vs. non-shockable rhythms).

The models will include relevant covariates, such as age, comorbidities, and other baseline characteristics, to adjust for potential confounders. Random effects will be included to account for variability between EMS regions.

### Additional statistical considerations

All analyses will be conducted on an intention-to-treat basis. Exploratory subgroup analyses are planned to evaluate potential effect modifications, including presumed etiology of OHCA (respiratory/asphyxial vs. primary cardiac) and airway management difficulty. These analyses will be hypothesis-generating and interpreted with caution.

The study hypothesis is that ventilation with PEEP (5 mbar) increases the probability of ROSC compared with ZEEP (0 mbar).

No interim analyses are planned.

### Interim analyses {21b}

Interim analyses are not planned during the 1-year study phase.

### Methods for additional analyses (e.g., subgroup analyses) {20b}

Exploratory subgroup analyses are planned to evaluate potential effect modifications related to the study intervention.

The presumed etiology of OHCA — categorized as respiratory/asphyxial versus primary cardiac — will serve as a key subgroup variable. This classification will be based on dispatcher information, scene findings, and the EMS team’s clinical impression.

In addition, the eCRF systematically records a broad range of parameters that allow for further exploratory analyses, including:

- Patient comorbidities (e.g., cardiac or pulmonary disease)
- Airway management characteristics (type of device, number of attempts, complications)
- Ventilation parameters (PEEP, FiO₂, etCO₂, SpO₂, pCO₂)

These variables will enable investigation of potential effect modifiers and interactions, such as which patient groups may particularly benefit from PEEP during resuscitation.

All subgroup analyses will be pre-specified, and interpreted with caution given the pilot nature of the study. The results will be considered hypothesis-generating to guide the design of future confirmatory research.

### Methods in analysis to handle protocol non-adherence and any statistical methods to handle missing data {20c}

Missing or incomplete data will be monitored continuously through automated completeness checks within the eCRF. In cases of protocol non-adherence or deviations in data entry, the coordinating center will contact the respective EMS team for clarification and data verification.

All missing data and deviations from the protocol will be documented and reported transparently.

For the statistical analysis, missing data will be handled using appropriate methods depending on the type and extent of missingness. If feasible, multiple imputation will be applied for missing covariate data. Sensitivity analyses will be conducted to evaluate the robustness of the primary results under different assumptions regarding missingness and protocol adherence.

This approach ensures transparency, minimizes potential bias, and maintains the methodological rigor of the study despite the operational challenges of data collection in the pre-hospital setting.

### Plans to give access to the full protocol, participant-leveldata and statistical code {31c}

Not applicable.

## Oversight and monitoring

### Composition of the coordinating center and trial steering committee {5d}

The coordinating center is comprised of a central team responsible for day-to-day management, operational support, and data coordination. This team ensures that all study procedures are implemented according to the protocol and meets regularly to monitor progress.

In addition, the trial steering committee provides overall oversight, strategic guidance, and ensures adherence to the study protocol. This committee meets at predetermined intervals throughout the trial to review progress and address any issues. Supporting groups, such as the endpoint adjudication committee and the data management team, are also involved in maintaining data quality and trial integrity.

It is important to note that no interim analyses are planned during the course of the trial.

### Composition of the data monitoring committee, its role and reporting structure {21a}

In this trial, a formal Data Monitoring Committee (DMC) has not been established. Given the pilot nature of the study, the limited risk associated with the interventions—which consist of standard resuscitation practices—and the absence of planned interim analyses, a dedicated DMC is not deemed necessary. Instead, oversight of the trial is provided by the coordinating center and the trial steering committee, which meet regularly to monitor safety, data quality, and overall study progress. These groups operate independently from the sponsor and any potential competing interests, ensuring that safety concerns are promptly addressed. Further details regarding trial oversight can be found in the trial’s governance documentation.

### Adverse event reporting and harms {22}

All adverse events (AEs) and unintended effects of the trial interventions will be systematically collected, assessed, reported, and managed. Both solicited events (actively monitored) and spontaneously reported events by the emergency team will be documented using standardized reporting forms within the eCRF. Each event will be evaluated for severity, causality, and expectedness by the designated study team. Serious adverse events (SAEs) will be reported promptly to the trial steering committee and the ethics committee, and managed according to established protocols. Regular reviews of AE data will be conducted to ensure ongoing participant safety and, if necessary, to inform modifications to the trial conduct. Comprehensive details on the adverse event management process are provided in the trial’s standard operating procedures.

### Frequency and plans for auditing trial conduct {23}

N/a.

### Plans for communicating important protocol amendments to relevant parties (e.g., trial participants, ethical committees) {25}

In the event that amendments are necessary, both the ethics committee and the participating centers will be informed. The participating centers will then also receive additional training.

### Dissemination plans {31a}

After the study is completed, a central evaluation of the collected data will be carried out. The participating centers will then receive individualized data for their respective study center. However, the publication of the overall data will be done collectively for all participating sites.

## Discussion

In recent decades, numerous studies have demonstrated the importance of high-quality chest compressions and early defibrillation for the prognosis of patients requiring resuscitation.

While the implementation of optimal initial airway management using ETI or EGA has been the subject of numerous studies and controversial discussions in recent years, there is surprisingly little evidence with regard to the optimal ventilation strategy for patients following airway management. This is all the more astonishing as, due to the continuously decreasing PaO_2_ during circulatory arrest, ventilation obviously becomes more important to ensure tissue oxygenation as the duration of resuscitation increases.

Animal studies have shown that after induction of circulatory arrest, the cessation of spontaneous breathing as well as mechanical chest compressions cause a large part of the lung to exhibit relevant atelectasis (in up to 73% of the lung surface), which could impair gas exchange on the one hand and hemodynamics on the other. Although a favorable effect on prognosis has been demonstrated for the application of a FiO_2_ of 1.0 during resuscitation of adult patients, there is no evidence to date for the oxygenation factor of PEEP, which is particularly important in intensive care medicine. The application of PEEP during resuscitation could improve DO2 and thus the oxygenation of organs at risk, such as the brain, by recruiting and keeping previously atelectatic lung areas open, and could also have a positive effect on hemodynamics.

This study faces several practical and operational challenges related to its conduct in a pre-hospital emergency setting. One key issue is the logistics of enrolling patients during OHCA, where time-sensitive decisions must be made. Ensuring that EMS personnel adhere to the study protocol while delivering urgent care will require thorough training and clear communication. Another challenge is the cluster-randomized design, which involves different EMS regions rotating between the PEEP and ZEEP groups. This design requires careful coordination and monitoring to ensure that randomization is properly implemented and that no unintentional crossover occurs between groups. To ensure this, it is planned to program the emergency ventilation modes of the ventilators according to cluster randomization. In addition, regular training sessions and stickers placed on the ventilators are intended to make rescue service staff aware of which group the patient is in at the time of resuscitation.

Additionally, data collection during such high-pressure, fast-paced environments may be difficult. Ensuring accurate and complete documentation of ventilation settings and patient outcomes will be critical. For this reason, it is planned to document the measures taken following the operation using an application-based eCRF. In addition, the emergency respirators will be read out after use so that real-life data on the ventilation settings measured by the respirators is available.

Moreover, variability in care practices across EMS regions may introduce confounding factors that will need to be accounted for in the statistical analysis.

However, the treatment of resuscitation is highly standardized by the regularly updated resuscitation guidelines and the rescue service staff in Germany are regularly re-certified in the performance of CPR in accordance with their legally prescribed further training obligation.

These issues, along with the ethical considerations of conducting research in life-threatening situations, will need careful attention throughout the study.

## Trial status

This is the second version of the protocol. Recruitment commenced on June 1, 2025, and is expected to be completed by May 31, 2026. The study is currently ongoing across the participating emergency medical service (EMS) regions.

## Data Availability

The principal investigator will have access to the anonymized final trial dataset. The other study coordinators have access to the data records in their respective study centers.

## Abbreviations

CRF: Case report form
DGAI: German Society for Anaesthesiology, Intensive Care Medicine, Emergency Medicine, and Pain Therapy
EAD: Extraglottic airway device
eCRF: Electronic Case Report Form
EKG: Electrocardiogram
EMS: Emergency medical services
etCO2: End-tidal carbon dioxide
ETT: Endotracheal tube
FiO2: Fraction of inspired oxygen
GCS: Glasgow coma scale
HR: Heart rate
ICU: Intensive care unit
OHCA: Out-of-hospital cardiac arrest
PEEP: Positive end-expiratory pressure
ROSC: Return of spontaneous circulation
SBP: Systolic blood pressure
SpO2: Pulse oximetry oxygen saturation
ZEEP: Zero end-expiratory pressure
